# Ciliate Epibionts Associated with Crustacean Zooplankton in German Lakes: Distribution, Motility, and Bacterivory

**DOI:** 10.3389/fmicb.2012.00243

**Published:** 2012-07-05

**Authors:** Samantha L. Bickel, Kam W. Tang, Hans-Peter Grossart

**Affiliations:** ^1^Virginia Institute of Marine Science, College of William and MaryGloucester Point, VA, USA; ^2^Department of Limnology of Stratified Lakes, Leibniz-Institute of Freshwater Ecology and Inland FisheriesStechlin, Germany; ^3^Institute for Biochemistry and Biology, Potsdam UniversityPotsdam, Germany

**Keywords:** ciliate epibionts, *Epistylis*, crustacean zooplankton, bacterivory, epibiont motility

## Abstract

Ciliate epibionts associated with crustacean zooplankton are widespread in aquatic systems, but their ecological roles are little known. We studied the occurrence of ciliate epibionts on crustacean zooplankton in nine German lakes with different limnological features during the summer of 2011. We also measured the detachment and re-attachment rates of the ciliates, changes in their motility, and the feeding rates of attached vs. detached ciliate epibionts. Epibionts were found in all lakes sampled except an acidic lake with large humic inputs. Epibiont prevalence was as high as 80.96% on the cladoceran *Daphnia cucullata*, 67.17% on the cladoceran *Diaphanosoma brachyurum*, and 46.67% on the calanoid copepod *Eudiaptomus gracilis*. Both cladoceran groups typically had less than 10 epibionts per individual, while the epibiont load on *E. gracilis* ranged from 1 to >30 epibionts per individual. After the death of the zooplankton host, the peritrich ciliate epibiont *Epistylis* sp. detached in an exponential fashion with a half-life of 5 min, and 98% detached within 30 min, leaving behind the stalks used for attachment. Immediately after detachment, the ciliates were immotile, but 62% became motile within 60 min. When a new host was present, only 27% reattached after 120 min. The average measured ingestion rate and clearance rate of *Epistylis* were 11,745 bacteria ciliate^−1^ h^−1^ and 24.33 μl ciliate^−1^ h^−1^, respectively. Despite their high feeding rates, relatively low epibiont abundances were observed in the field, which suggests either diversion of energy to stalk formation, high metabolic loss by the epibionts, or high mortality among the epibiont populations.

## Introduction

Both free-swimming and attached ciliates play key roles in freshwater and marine food webs (Sherr and Sherr, 1987; Sanders et al., 1989; Carrias et al., 1996). Although attached ciliates are very common in the benthos (Borror, 1968; Fenchel, 1969), their presence is not limited to bottom substrates. Many ciliates and other protozoans can attach themselves to various surfaces among the plankton such as suspended particles, phytoplankton, and zooplankton (Fernandez-Leborans and Tato-Porto, 2000; Christensen-Dalsgaard and Fenchel, 2003; Šimek et al., 2004). Attachment to a surface that experiences increased flow can enhance feeding rates by reducing the boundary layer surrounding the protozoan (Shimeta et al., 2001). In addition, because planktonic ciliates use their cilia both to generate thrust for swimming and to create a feeding current, attachment to a surface may help balance the thrust with drag and direct the flow field toward the cells, thereby increasing their food capturing efficiency relative to free-swimming individuals (Christensen-Dalsgaard and Fenchel, 2003). These predictions have been experimentally verified for flagellates (Christensen-Dalsgaard and Fenchel, 2003) and ciliates (Shimeta et al., 2001; Jonsson et al., 2004). Consequently, in systems where attached ciliates and other protozoans are abundant, they may contribute substantially to the total grazing impact. For example, attached flagellates on diatom colonies have been reported to account for up to 64% of all bacterivory by protists in an oligo-mesotrophic lake (Carrias et al., 1996) and a meso-eutrophic reservoir (Šimek et al., 2004).

While some ciliates may attach and detach in a haphazard manner that requires no special adhesive mechanism (Jonsson et al., 2004), others use distinct and elaborate structures, such as the stalks in peritrich ciliate epibionts, to more firmly attach to surfaces (Randall and Hopkins, 1962). The production of stalks and the subsequent loss of these structures during detachment represent a considerable energy investment by the ciliates, and must be compensated by substantial benefits of attachment. Based on previous studies with benthic ciliates (Shimeta et al., 2001) and stalk-less ciliate epibionts (Jonsson et al., 2004), one expects that stalked ciliates will have much higher feeding rates than their free-swimming form, although such a direct comparison is rarely made.

Within the aquatic environments, crustacean zooplankton such as copepods and cladocerans are the dominant members of the zooplankton community. Their exoskeleton provides abundant surfaces for attachment by a wide range of organisms, including bacteria, fungi, algae, and protozoans (Carman and Dobbs, 1997). Stalked ciliate epibionts on freshwater and marine crustacean zooplankton have been documented in many parts of the world (e.g., Fernandez-Leborans and Tato-Porto, 2000; Puckett and Carman, 2002; Cabral et al., 2010; Rajabunizal and Ramanibai, 2011). Most studies tend to focus on the adverse effects these epibionts have on the host, such as decreased fecundity, interference with feeding, and locomotion, and increased sensitivity to contaminants (Kankaala and Eloranta, 1987; Puckett and Carman, 2002; Gilbert and Shröder, 2003). In comparison, quantitative information about the distribution and trophic impacts of these epibionts remains scarce (Utz and Coats, 2005a). Stalked epibiont ciliates exhibit two distinct life stages: the attached trophont stage for feeding, and the free-swimming telotroch stage for dispersal. Free-swimming telotrochs can result from asexual reproduction or through detachment and transformation of trophonts (Gilbert and Shröder, 2003). The transition from trophonts to telotrochs appears to be triggered by molting or death of the zooplankton host (Green, 1974; Willey and Threlkeld, 1995; Utz and Coats, 2008). Understanding the transition from attached to free-swimming form and vice versa, as well as the behaviors of each life stage, will help elucidate the life history and ecology of these organisms (Utz and Coats, 2008).

The northeastern region of Germany contains many glacially formed lakes with different nutrient conditions that are representative of lakes in other temperate regions. Many of them are important for inland fisheries, tourism, and navigation. While the occurrence of ciliate epibionts on crustacean zooplankton has been observed in some of these lakes (P. Kasprzak personal communication), no quantitative information is available. Our objective was to study the prevalence and abundance of ciliate epibionts attached to crustacean zooplankton in nine freshwater lakes with different limnological features, but located within a spatially small area in this region. Several of these lakes are part of a long-term monitoring program, and one of them, Lake Stechlin, is also a member of the international Global Lake Ecological Observatory Network. Additionally, we observed the behavior of the common peritrich ciliate epibiont, *Epistylis* sp., including its rate of detachment after host death, its motility after detachment, and its re-attachment to new hosts. We also measured rates of bacterivory by both attached and free-living stages of *Epistylis* sp. in laboratory experiments to estimate the potential importance of ciliate epibionts as bacterivores within these lakes.

## Materials and Methods

### Field sampling

**Figure 1:**
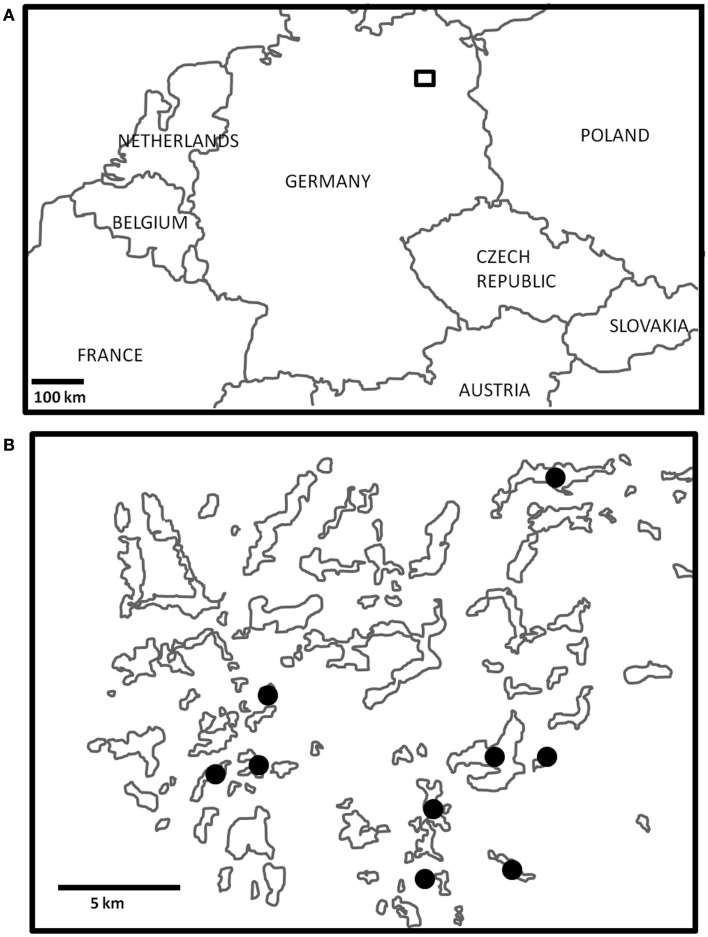
**(A)** The general study region in northeast Germany is outlined by the small square box, which is enlarged in **(B)**. Three Lakes sampled are denoted by black dots.

Zooplankton samples were collected from nine lakes in Northeast Germany within a 20-km radius (Figure 1) over a 2-week period in July 2011. The lakes sampled were all glacially formed, and encompass a wide range of sizes, depths, and nutrient conditions (Table 1). Triplicate samples were collected from each lake by vertical tows with a 90-μm mesh plankton net equipped with a filtering cod end, immediately transferred to 100-ml glass jars, and preserved with Lugol’s Iodine solution. Towed volumes were calculated based on the mouth diameter of the net (58 cm) and towed depths. In the laboratory, 5–15 ml subsamples were examined for zooplankton species composition, ciliate epibiont prevalence (the percent of a zooplankton group with ciliate epibionts), and epibiont load (the number of ciliate epibionts per individual zooplankter). Counts from the subsamples were extrapolated to the entire sample, and the average of the three replicates from each site was calculated. These values were used to estimate total epibiont densities (the number of epibionts per cubic meter) in each lake.

**Table 1:** **Limnological characteristics for sampled lakes**.

Lake	Area (km^2^)	Max depth (m)	Nutrient condition	Latitude and longitude
Dagow	0.22	8	Eutrophic	53°09′N, 13°03′E
Stechlin	4.30	68	Oligotrophic	53°09′N, 13°01′E
Grosse Fuchskuhle	0.08	5.6	Eutrophic	53°10′N, 13°02′E
Nehmitz	1.60	18.6	Mesotrophic	53°08′N, 12°59′E
Roofen	0.57	19.1	Mesotrophic	53°06′N, 13°02′E
Drewen	2.56	9	Eutrophic	53°15’N, 13°03’E
Prebelow	2.80	7.6	Eutrophic	53°10’N, 12°52’E
Schlaborn	0.70	9	Eutrophic	53°09′N, 12°52′E
Dollgow	0.18	2	Eutrophic	53°04′N, 13°00′E

### Epibiont detachment, motility, and re-attachment observations

While epibionts were found on a number of zooplankton species, the calanoid copepod *Eudiaptomus gracilis* with the ciliate epibiont *Epistylis* sp. was the most common in the samples, and was subsequently used for all laboratory experiments. *Eudiaptomus gracilis* was collected from Lake Dagow and transported back to the lab in ambient water. Copepods carrying large numbers of the ciliate epibiont *Epistylis* sp. were sorted into 5 μm-filtered surface lake water. Individual copepods were then transferred to a hanging drop slide with a small drop of the surrounding water. The copepod was killed by crushing its cephalosome with a fine-tipped forceps and the total number of epibionts initially attached to the copepod was immediately counted using a dissecting microscope. The number of epibionts that remained attached to the copepod carcass was counted every 5 min for up to 40 min after copepod death. Ten replicates were performed.

In a second experiment, copepods with attached *Epistylis* sp. were isolated and killed as described above. The copepod carcasses were removed after 30 min, and the motility of detached ciliates was observed every 20 min for 2 h. After the 2-h observation, a new copepod without epibionts was added to the ciliates. To allow the new copepod to move freely, the total volume was adjusted to 350 μl with 0.2 μm-filtered lake water. The copepod was visually inspected every 20 min and the number of reattached ciliates was estimated. After 2 h, the copepod was preserved with Lugol’s Iodine solution and the actual number of attached ciliate epibionts was counted. Ten replicates were performed. All detachment, motility, and re-attachment observations were performed at room temperature (23°C).

### Bacterivory experiments

The calanoid copepod *Eudiaptomus gracilis* and surface water were collected from Lake Stechlin and Lake Dagow, and transported back to the laboratory. In the laboratory, copepods without epibionts and copepods with a large number of epibionts were picked from the field samples and rinsed in 0.2 μm-filtered water from the same lakes to remove detritus. Ten millilitres of diluted lake water (1:50 and 1:100 for Lake Stechlin and Lake Dagow, respectively) were added to 25-ml sterilized glass vials. Dilutions were prepared by adding 5 μm-filtered lake water to 0.2 μm-filtered lake water. Four sets of vials, each set with five replicates, were prepared as follows: (1) vials for enumerating initial bacterial abundance, (2) control vials without copepods to account for bacterial growth during the incubation, (3) experimental vials for copepods without epibionts, and (4) experimental vials for copepods with epibionts. Three copepods were gently pipetted into each of the experimental vials, with minimal surrounding water. Copepod addition was mimicked in the initial and control vials. Vials for initial bacterial abundance were processed immediately; all other vials were incubated for 2 h at room temperature (23°C). To measure free-living bacterial abundance, vials were swirled gently to mix the water; a 2-ml aliquot was removed without removing the copepods, and filtered onto a 0.2 μm polycarbonate membrane filter. Samples were stained with SYBR-gold nucleic acid stain in Citifluor and counted on an epifluorescent microscope with blue light excitation. Vials containing copepods were preserved with Lugol’s iodine solution and the total number of ciliate epibionts in each vial was counted.

To measure the grazing rates of free-swimming ciliate epibionts, copepods carrying *Epistylis* sp. were collected from Lake Dagow and sorted into 0.2 μm-filtered surface lake water. Individual copepods were killed as described previously, and the ciliates were allowed to detach for 30 min. Afterward, the copepod carcass was removed, and 50–56 free-swimming ciliates were transferred to each of the experimental vials containing 10 ml of diluted Lake Dagow water, prepared as described previously. Three sets of vials, each set with five replicates, were established as follows: (1) vials for initial bacterial abundance, (2) control vials without ciliates, and (3) experimental vials with free-swimming ciliates. The vials were incubated at room temperature for 2 h. Bacterial abundance was measured as described previously. Bacterial abundance data were tested for normality and differences among the treatments were assessed with a one-way ANOVA followed by *post hoc* Tukey pairwise comparisons. When a significant change in bacterial abundance was observed, ingestion and clearance rates were calculated after correcting for changes in bacterial abundances in the control and epibiont-free copepod treatments.

## Results

### Field study

**Figure 2:**
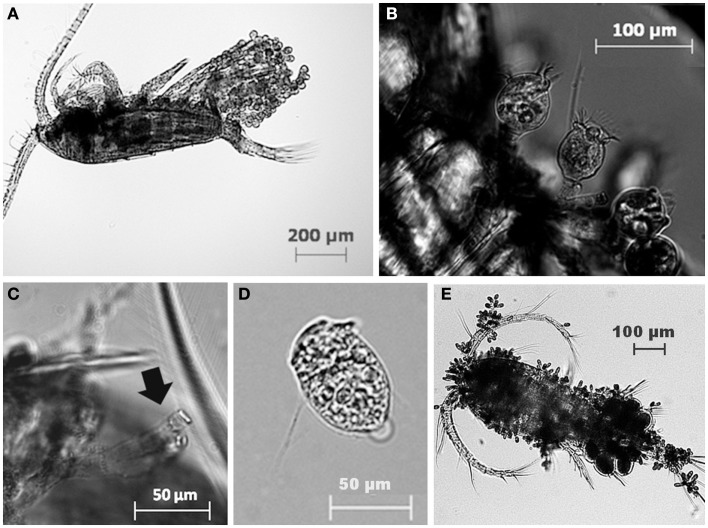
**Examples of the peritrich ciliate epibiont *Epistylis* sp. attached to the calanoid copepod *Eudiaptomus gracilis* (A,B) and the residual stalk (C) left attached to the dead copepod after detachment (D)**. Peritrich ciliates of the family Opercularidae, attached to a cyclopoid copepod **(E)**.

**Figure 3:**
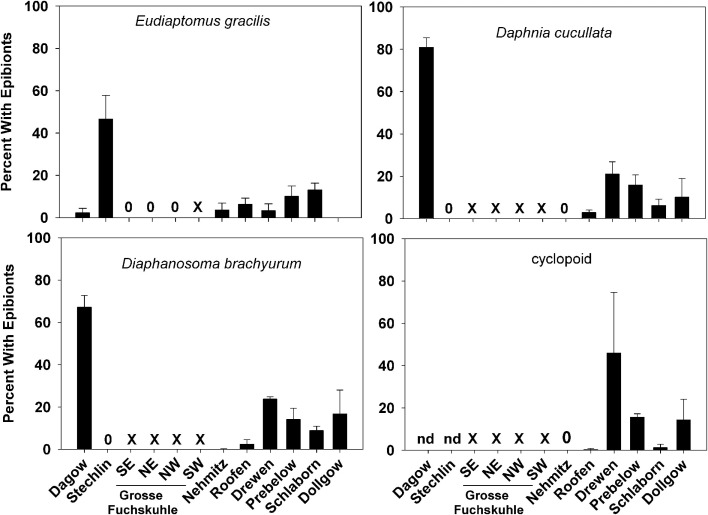
**Prevalence of ciliate epibionts (percent of the zooplankton population with one or more ciliate epibionts; mean ± SD, *n* = 3) on common zooplankters *Eudiaptomus gracilis, Daphnia cucullata*, *Diaphanosoma brachyurum*, and Cyclopoid copepods in each lake**. 0 = no epibionts were found on that zooplankton group, X = zooplankton group was not present in that lake, nd = no data.

The most common zooplankton species found among the lakes were the calanoid copepod *Eudiaptomus gracilis* and the cladocerans *Daphnia cucullata* and *Diaphanosoma brachyurum* (Table 2). Cyclopoid copepods were also present, but were not identified to any lower taxonomic level. A variety of epibionts, including diatoms and ciliates, were found in association with zooplankton in the lakes. Ciliate epibionts were identified according to criteria outlined by Patterson and Hedley (2003). The mobile peritrich ciliate *Trichodina* sp. was commonly observed on live copepods from Lake Dagow and Lake Stechlin; however, it immediately detached upon preservation, and therefore was not included in the data. The stalked peritrich ciliate *Vorticella* sp. was occasionally observed on cladocerans. The most common epibiont carried by *E. gracilis*, *D. cucullata*, and *D. brachyurum* was identified as the peritrich ciliate *Epistylis* sp. based on the stalk branching pattern, stalk thickness relative to cell size, and the non-contractile nature of the stalks (Figures 2A–D). Cyclopoid copepods occasionally carried *Epistylis* sp., but the majority of epibionts carried by cyclopoids were peritrich ciliates belonging to the family Opercularidae (Figure 2E). The prevalence of epibionts was highly variable within a single species among the different lakes, and among different species within the same lake. Among the sampled lakes, epibiont prevalence on *E. gracilis* ranged from 0% (Lake Dollgow) to 46.67% (Lake Stechlin) of the population (Figure 3). Epibiont prevalence ranged between 0% (Lakes Stechlin and Nehmitz) and 80.96% (Lake Dagow) on *D. cucullata* and between 0% (Lake Stechlin) and 67.16% (Lake Dagow) on *D. brachyurum* (Figure 3). Epibionts were found on at least one zooplankton group in all lakes sampled except Lake Grosse Fuchskuhle.

**Table 2:** **Mean (SD) densities of common zooplankton species and their associated ciliate epibionts in the lakes studied**.

			Lake											
			Dagow	Stechlin	Grosse Fuchskuhle				Nehmitz	Roofen	Drewen	Grosser Prebelow	Schlaborn	Dollgow
					SE	NE	NW	SW						
**DENSITY: INDIVIDUALS m^−3^ (MEAN SD)**														
*E. gracilis*	Zooplankter		2656 (418)	392 (61)	18,542 (3278)	10,168 (3780)	10,713 (2577)	0(0)	1972 (145)	977 (132)	382 (10)	492 (157)	522 (206)	0 (0)
	Epibiont		56,092 (77,605)	2823 (1067)	0 (0)	0 (0)	0 (0)	0(0)	1031 (722)	990 (1057)	144 (215)	968 (430)	1018 (155)	0 (0)
*D. cucullata*	Zooplankter		1937 (641)	1129 (272)	0 (0)	0 (0)	0 (0)	0(0)	668 (45)	1368 (214)	778 (181)	1077 (226)	2327 (382)	686 (172)
	Epibiont		17,303 (5252)	0 (0)	0 (0)	0 (0)	0 (0)	0(0)	0 (0)	348 (149)	1173 (504)	559 (227)	540 (115)	381 (449)
*D. brachyurum*	Zooplankter		1169 (111)	109 (53)	0 (0)	0 (0)	0 (0)	0(0)	2235 (589)	579 (112)	1197 (269)	2528 (205)	782 (98)	398 (87)
	Epibiont		642,726 (258,241)	0 (0)	0 (0)	0 (0)	0 (0)	0(0)	22 (38)	103 (90)	2776 (904)	1444 (664)	430 (146)	1130 (570)
Cyclopoid	Zooplankter		nc	nc	0 (0)	0 (0)	0 (0)	0(0)	255 (220)	919 (56)	2140 (76)	3009 (853)	2185 (411)	2349 (1088)
	Epibiont		nc	nc	0 (0)	0 (0)	0 (0)	0(0)	0 (0)	115 (199)	38,193 (13,257)	7261 (3812)	12 (21)	8508 (3462)
*Bosmina*	Zooplankter		nc	nc	0 (0)	0 (0)	0 (0)	0 (0)	942 (152)	696 (157)	106 (37)	1208 (214)	1903 (204)	170 (27)
	Epibiont		nc	nc	0 (0)	0 (0)	0 (0)	0 (0)	0 (0)	0 (0)	55 (95)	188 (32)	0 (0)	0 (0)
*Ceriodaphnia*	Zooplankter		nc	nc	9297 (3918)	6388 (2798)	11,394 (3889)	53,752 (9326)	77 (53)	0 (0)	0 (0)	0 (0)	0 (0)	0 (0)
	Epibiont		nc	nc	0 (0)	0 (0)	0 (0)	0(0)	0 (0)	0 (0)	0 (0)	0 (0)	0 (0)	0 (0)
**EPIBIONT BACTERIVORY**														
(10^8^) Bacteria consumed (m^−3^ day^−1^)			75.2	8.4	0	0	0	0	3.37	4.77	122	34.1	6.51	30.1
% Bacterial standing stock consumed			0.189	0.021	0	0	0	0	0.008	0.012	0.304	0.085	0.016	0.075

*Lake Grosse Fuchskuhle is divided into four basins (SE, NE, NW, and SW). The estimated number of bacteria grazed by all ciliate epibionts and the percentage of bacterial standing stock consumed by ciliate epibionts within each lake are also presented. For bacterivory calculations, the ingestion rate used was the average between Lake Stechlin and Lake Dagow. An ambient bacterial abundance of 4 × 10^6^ cells ml^−1^ was assumed*.

*Epibiont = ciliate epibionts associated with that particular zooplankter per m^−3^*.

*nc = not counted*.

**Figure 4:**
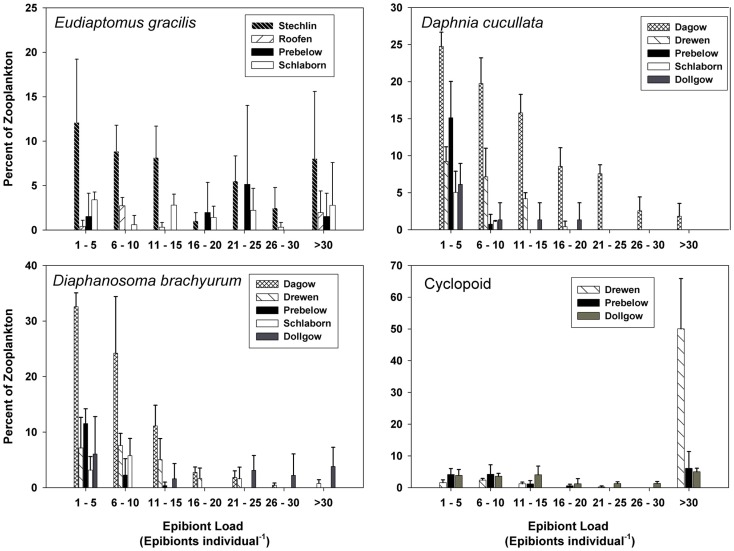
**Frequency distribution of epibiont load (number of epibionts per individual, mean ± SD, *n* = 3) on *E. gracilis, D. cucullata*, *D. brachyurum*, and cyclopoid copepods**. Epibiont load is presented only for lakes where epibionts were found on >5% of the zooplankton populations.

Of the various zooplankton species where at least 5% of the population carried one or more epibionts, species-specific frequency distributions of epibiont load were noted. The epibiont load on *E. gracilis* ranged from 1 to >30, and the frequency distribution was fairly uniform in each of the lakes (Figure 4). The two cladoceran species, *D. cucullata* and *D. brachyurum*, showed a similar range in epibiont load (1 to >30); however, the frequency distribution was skewed toward the lower end such that the majority of cladocerans carried ≤15 epibionts per individual (Figure 4). The epibiont loads on cyclopoid copepods were also fairly uniformly distributed, with the exception of Lake Drewen, in which 50.02% of cyclopoids carried >30 epibionts per individual (Figure 4). The estimated densities of epibionts in the lakes are presented in Table 2.

### Epibiont detachment, motility, and re-attachment

**Figure 5:**
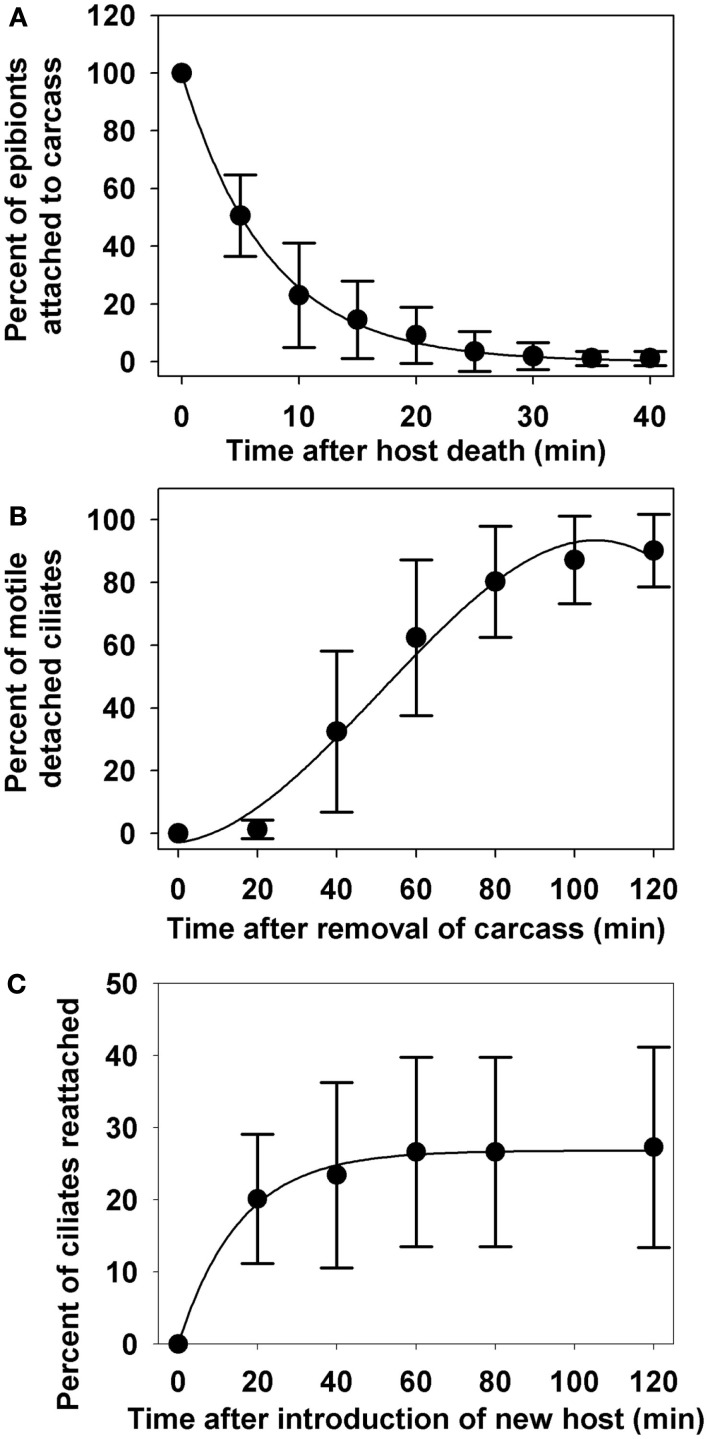
**Percentage of epibionts remaining on zooplankton host, *E. gracilis* after host death (A), motility of epibionts detached from copepod carcasses (B), and re-attachment of free-swimming ciliates to new copepod hosts (C)**. All data points are mean values ± SD (*n* = 10).

After the death of the copepod host, 49.4% of all epibionts detached within the first 5 min. During detachment the ciliate exhibited a rocking motion to break free from the stalk, leaving the stalk attached to the copepod host (Figures 2C,D). Immediately after detachment, the ciliates were either immobile or swimming very slowly in tight circular patterns. After 30 min, the copepod carcass was removed; 98.1% of the epibionts had detached (Figure 5A) and observations of their motility continued. The detachment of epibionts from copepod carcasses was well described (*R*^2^ = 0.998, *p* < 0.0001) by the exponential decay function:

$$y_{1}=99.7e^{-0.136x}$$

where y_1_ is percent of epibionts that remained on the copepod and x is time in minutes. Twenty minutes after the removal of copepod carcass (i.e., 50 min. after host death), only 1.28% of the detached ciliates were active and rapidly swimming. Motility increased to 62.14% by 60 min after carcass removal, and 90.18% by 120 min after carcass removal (Figure 5B). The percentage of detached ciliates actively swimming (y_2_) as a function of time (x; minutes) could be described (*R*^2^ = 0.986, *p* = 0.0027) by the polynomial equation:

$$y_{2}=-2.811+0.1561x+0.0231x^{2}-0.0002x^{3}$$

When a new copepod host was introduced, 20.13% of the free-swimming ciliates attached to the new host within the first 20 min, but re-attachment leveled off at 27.27% after 120 min (Figure 5C). The percentage of reattached ciliates (y_3_) as a function of time (x) was well represented (*R*^2^ = 0.995, *p* < 0.0001) by the equation:

$$y_{3}=26.83\left(1-e^{-0.65x}\right)$$

### Bacterivory rates

**Figure 6:**
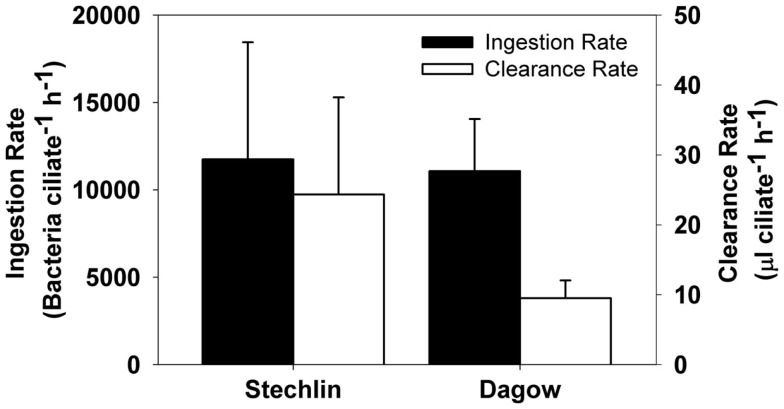
**Estimated ingestion rates (black bars, left axis) and clearance rates (white bars, right axis) of the peritrich ciliate *Epistylis* sp. when attached to the calanoid copepod *Eudiaptomus gracilis* collected from Lake Stechlin and Lake Dagow (mean ± SD, *n* = 5)**.

In the first experiment conducted with the copepod *E. gracilis*, there was a significant increase in bacterial abundance in both the control vials and copepod vials (*p* < 0.001), but the final bacterial abundance was significantly lower in vials with copepods that carried attached epibionts (*p* < 0.001), indicating grazing activities by the epibionts. Ingestion rates were 11,745 ± 6701 and 11,065 ± 2986 (mean ± SD) bacteria ciliate^−1^ h^−1^ for Lake Stechlin and Lake Dagow, respectively (Figure 6). The corresponding clearance rates were 24.33 ± 13.88 and 9.49 ± 2.56 (mean ± SD) μl ciliate^−1^ h^−1^, respectively. In the second experiment conducted with free-swimming ciliate epibionts, there was no significant change in the bacterial abundance in either the control vials or vials with ciliates, indicating that grazing activity by free-swimming epibionts was negligible.

## Discussion

### Epibiont abundance and distribution

The peritrich ciliate *Epistylis* sp. observed in our study exhibited a low degree of host specificity and colonized both calanoid copepods and cladocerans, consistent with other studies (Green, 1974; López et al., 1998; Gilbert and Shröder, 2003). Despite the close proximity and sampling period of the different lakes, there were large inexplicable differences in the prevalence of epibionts between the different zooplankton species within the same lake, and between the different lakes for the same zooplankton species. For example, both cladocerans *D. cucullata* and *D. brachyurum* were heavily infested (>60% of the population) in Lake Dagow, but no epibionts were found on these species in Lake Stechlin. In contrast, 46% of the copepod *E. gracilis* carried epibionts in Lake Stechlin but less than 5% had epibionts in Lake Dagow. Because both cladocerans and copepods are covered by chitinous carapace, it is unlikely that the different prevalence was a result of differences in body surface chemistry. In laboratory experiments, Gilbert and Shröder (2003) showed that *Epistylis pygmaeum* preferentially attached to some zooplankton species but not others, but the cause remained unknown. Although Lake Dagow and Lake Stechlin are physically connected by a small drainage canal, they are very different environments: Lake Dagow is a shallow, eutrophic lake while Lake Stechlin is a deep, oligotrophic lake. The different environmental conditions between the lakes may modulate epibiont attachment to the different zooplankton species (Threlkeld et al., 1993).

Overall, the ciliate epibiont prevalence observed in our study was similar to that reported in other field studies. For examples, approximately 35% of Chironomid larvae carried the ciliate epibiont *Rhabdostyla* in a tropical lake system (Cabral et al., 2010), 48% of the calanoid copepod *Metridia pacifica* collected from the northeast Pacific Ocean were infested with suctorian ciliates (Ohtsuka et al., 2011), 50% of the Harpactacoid copepod *Coullana* spp. from a salt marsh carried peritrich ciliates (Puckett and Carman, 2002), and up to 87% of rotifers maintained in culture supported *Epistylis* sp. (Gilbert and Shröder, 2003). In our study, Lake Grosse Fuchskuhle was the only lake in which no epibionts were found on any zooplankton species. Lake Grosse Fuchskuhle is an acidic lake with large humic inputs (average pH = 4.2–4.6; Casper et al., 2003), whereas the average pH in the other lakes is slightly basic (e.g., average pH during the study period = 8.1 in Lake Dagow and Lake Stechlin). This suggests that the acidic environment in Lake Grosse Fuchskuhle is unfavorable to the epibionts.

### Epibiont behavior

The rapid detachment after death of the zooplankton host suggests that the ciliate epibiont was able to quickly detect a change in the host condition that made attachment no longer beneficial. Utz and Coats (2005b) suggested that some chemical or electrical cue may travel through the stalk of the epibiont to the zooid. Changes in these cues may occur during molting or after death, and trigger detachment. In this study, the number of epibionts attached to *E. gracilis* decreased exponentially after host death. This same behavior was observed in the marine ciliate epibiont *Zoothamnium intermedium* detaching from the calanoid copepods *Acartia tonsa* and *Eurytemora affinis*, but at different rates (Utz and Coats, 2008). The epibionts in our study had a detachment “half-life” of 5 min; i.e., approximately 50% of the epibionts detached within 5 min of host death and 98% detached within 30 min. In contrast, *Z. intermedium* detached at a much lower rate: 50 and 90% after 3 and 7 h after host death, respectively (Utz and Coats, 2008). Even chemically induced detachment occurred over a longer time period, with 80% of *Vorticella* detaching within 90 min (de Baufer et al., 1999). Re-attachment of the free-swimming ciliates to a new host also occurred on a shorter time scale in our experiments: 27% of the free-swimming ciliates attached within 2 h of introduction of a new host. In comparison, only ~15% of *Z. intermedium* attached within 2 h (Utz and Coats, 2008). The different detachment and re-attachment rates may reflect the extent to which the different ciliate species have adapted to a benthic life style among the plankton.

After detachment, the ciliates initially showed no or little movement, and only gradually regained full motility (Figure 5B). Gilbert and Shröder (2003) observed two distinct swimming patterns among different life stages of free-swimming *E. pygmaeum*: (i) detached zooids swimming slowly in a circular pattern, and (ii) fast, erratic-swimming telotrochs; they suggested that the *Epistylis* was able to switch back and forth between the two forms. Both swimming patterns were noted in our study, with a clear delay between the occurrence of the two different motilities, which may be a result of the time needed for physiological changes during the transformation from attached zooid to free-living zooid and/or telotroch. It has been suggested that the fast-swimming telotroch form does not feed and primarily functions as a way to find new hosts, whereas the slower, free-swimming zooid can feed and reproduce, allowing populations to persist when hosts are rare (Gilbert and Shröder, 2003).

The difference between detachment rate and re-attachment rate in our study is quite striking. While almost all epibionts detached within 40 min, less than 30% of them reattached even after 2 h (Figures 5A,C). Re-attachment was not limited by encounter probability because in our experiments, a new copepod host was exposed to an average ciliate density of 86 ciliates ml^−1^, and physical contact between copepod and free-swimming ciliates occurred frequently. However, most instances of physical contact did not result in attachment. Gilbert and Shröder (2003) suggested that attachment of *E. pygmaeum* is mediated by contact recognition of surface properties, which may be different on different body parts of the copepod. Local, small-scale water movement, such as the copepod feeding current, may also alter attachment efficiency.

Attachment is an adaptive behavior that can result in increased feeding rates (Christensen-Dalsgaard and Fenchel, 2003; Jonsson et al., 2004). However, attachment also has disadvantages because it restricts the movement of the ciliate, and exposes the ciliate to the same predation risks experienced by the host, or even increases predation risk of the host (e.g., Willey et al., 1990). For stalked epibionts such as the *Epistylis* species used in this study, more permanent attachment represents a significant energy investment in the form of stalk production. Using a stalk for attachment also further restricts the ciliate’s movement, putting it at an even greater risk than other more mobile epibionts, such as *Trichodina sp*. From a cost vs. benefit perspective, it is perhaps not surprising that re-attachment rate should be lower than detachment rate, as the ciliate would have to carefully select the right location for attachment, but it should quickly abandon the host under unfavorable condition (e.g., death of the host). The considerable energy investment for stalk production and the risks associated with attachment also implies that the ciliate must be able to extract substantial benefits for being attached. One such benefit is enhanced feeding efficiency as demonstrated in our bacterivory experiments.

### Bacterivory rates and feeding impacts

Significant feeding rates of the epibiont were measurable for the attached form, but not for the detached, free-swimming form. The short incubation time in our feeding experiments may have limited the feeding signal that we could detect with the free-swimming ciliates; nonetheless, our results indicate that the attached form had a much higher feeding efficiency than the free-swimming form of the epibiont. The estimated clearance rates of the attached epibiont were 9.49 ± 2.56 and 24.33 ± 13.88 (mean ± SD) μl ciliate^−1^ h^−1^ for Lake Dagow and Lake Stechlin, respectively. These were much higher than an expected clearance rate of ~0.5 μl ciliate^−1^ h^−1^ for free-living ciliates of similar size (ca. 45 μm equivalent spherical diameter (ESD; Fenchel, 1980). They were also higher than the reported maximum clearance rate of an *Epistylis* colony measured with monodisperse fluorescent latex beads (1.25 μl ciliate^−1^ h^−1^; Børsheim, 1984). However, the use of latex beads tends to underestimate protozoan feeding rate (Sherr et al., 1987), and a similarly high clearance rate has been observed for some ciliate species, e.g., *Lohmanniella spiralis* with a clearance rate of 24 μl ciliate^−1^ h^−1^ (Jonsson, 1986). The higher clearance rate of the attached form relative to the free-swimming form provides a clear benefit for epibiont attachment. The kinetics of small-scale water movement around a copepod and its abilities to deliver food and oxygen are an important factor in determining the distribution of ciliate epibionts on the copepod (Fernandez-Leborans et al., 2006). A marine benthic ciliate community can take advantage of the reduced boundary layer due to increased flow over the sediment surface and increase its clearance rate by a factor of 5 (Shimeta et al., 2001). The same concept can be applied to ciliate epibionts on zooplankton: As a zooplankter swims through the water, flow over the body surface increases, reducing the boundary layer, and allowing the epibionts to feed at a higher rate.

The estimated average ingestion rate of attached epibionts in our experiments was 11,745 bacteria ciliate^−1^ h^−1^, which is 1–3 orders of magnitude higher than that of common free-living bacterivorous ciliates (Sanders et al., 1989). Using fluorescent beads to investigate the ingestion rate of *Epistylis* within wastewater biofilms, Eisenmann et al. (2001) reported an ingestion rate as high as 1200 beads ciliate^−1^ h^−1^. Methodological differences may contribute to the difference in ingestion rate between our study and that by Eisenmann et al. (2001): The fluorescent bead technique measures what is actually ingested by the ciliates, whereas our incubation experiments measured what disappeared from the surrounding water. Increased ciliate filtration may have brought the bacteria in closer proximity to the copepod, where they could have attached to the copepod or ciliate stalks, thereby causing an overestimation of ingestion rates in our experiments. On the other hand, considering that the use of fluorescently labeled beads could underestimate bacterivory by a factor of 10 (Sherr et al., 1987), our measured ingestion rates were indeed comparable to that for *Epistylis* biofilms (Eisenmann et al., 2001).

Studies on the grazing impact of planktonic ciliates are often focused on free-living species (e.g., Fenchel, 1980), whereas the grazing impact of ciliate epibionts attached to zooplankton is rarely measured. Using our measured clearance rates and epibiont abundances for the various lakes, we estimated that bacterivory by zooplankton-associated ciliate epibionts in these lakes removes only 0.3% of the bacterial biomass present in the water column per day (Table 2). This small impact is a result of the low epibiont abundances within these lakes. Despite the low overall trophic impact, selective feeding by epibionts may influence the free-living bacterial community composition. Additionally, the high individual clearance rate and ingestion rate of the epibionts mean that each ciliate processed a large amount of bacterial biomass. Utz (2008) reported that at a food concentration of 10^6^ bacteria ml^−1^, comparable to our experimental food concentrations, the epibiont ciliate *Zoothamnium intermedium* attained a growth rate as high as 0.8 day^−1^. An even higher *in situ* growth rate (1.37 day^−1^) has been reported for benthic peritrich ciliate (Kusuoka and Watanabe, 1987). Our short incubation time did not allow us to measure growth. Nevertheless, if *Epistylis* sp. grows at a comparable rate in our lakes, its low *in situ* abundances would imply high mortality (Kusuoka and Watanabe, 1987). On the other hand, Sherr and Sherr (2002) calculated that a ciliate with an ESD of 40 μm feeding at a food concentration of 10 μg C L^−1^ would require a clearance rate of 20 μl cell^−1^ h^−1^ to support one cell doubling per day. The epibionts in our experiments had an ESD of 40–50 μm (excluding the stalk). Assuming a bacterial carbon content of 12.4 fg per cell (Fukuda et al., 1998), the food concentration in our experiments would be approximately 3.72 μg C L^−1^, which is less than the theoretical amount required to sustain one doubling per day. For stalked ciliates, growth may also manifest as stalk production in addition to cell multiplication. As the epibiont colony grows, the proportion of stalk biomass also increases. Using digitized images, we estimated that the biovolume ratio between the stalk and the cell ranged from 0.0097 for recently attached *Epistylis* to 0.44 for larger *Epistylis* colonies with elaborate stalk structures, and the average stalk-to-cell biovolume ratio was 0.18. As the stalk structure grows, it moves the ciliate farther away from the copepod surface, which may help increase feeding efficiency by reducing the formation of small-scale eddies near the surface (Pepper et al., 2010).

Alternative to being used for biomass production, ingested energy could be lost through respiration and excretion. The gross growth efficiency of free-living ciliates is ca. 0.4 (Hansen et al., 1997), hence, on average 60% of the ingested materials is respired or excreted. Assuming that the epibionts in our experiments had the same gross growth efficiency, their high feeding rate would translate to high respiration and excretion rates, making these epibiont colonies potential “hotspots” for remineralization within the water column.

In summary, epibiont ciliates were prevalent in all of the studied lakes except the acidic Lake Grosse Fuchskuhle. The high individual feeding rates but low abundances of the epibionts suggest that the epibiont populations were either controlled by high mortality or that a high percentage of the ingested bacterial biomass was remineralized. It is also possible that selective feeding by the epibionts may influence the free-living bacterial community in quality rather than quantity. Additional research into the population dynamics, grazing and bioenergetics of the epibionts will help resolve these questions and provide further insights into the ecological roles of these epibiont ciliates.

## Conflict of Interest Statement

The authors declare that the research was conducted in the absence of any commercial or financial relationships that could be construed as a potential conflict of interest.
